# Heterogeneity in fibroblast proliferation and survival in idiopathic pulmonary fibrosis

**DOI:** 10.3389/fphar.2014.00002

**Published:** 2014-01-23

**Authors:** David M. Habiel, Cory Hogaboam

**Affiliations:** Division of Pulmonary and Critical Care Medicine, Department of Medicine, Cedar Sinai Medical CenterLos Angeles, CA, USA

**Keywords:** Idiopathic pulmonary fibrosis (IPF), lung fibroblasts, myofibroblasts, Toll Like Receptor 9 (TLR9), C-C Chemokine receptor type 7 (CCR7), interleukin 6 (IL6), glycoprotein 130 (gp130), C-C motif chemokine ligand 21 (CCL21)

## Abstract

Idiopathic pulmonary fibrosis (IPF) is the most common form of interstitial lung disease characterized by the persistence of activated myofibroblasts resulting in excessive deposition of extracellular matrix proteins and profound tissue remodeling. Myofibroblasts have been shown to arise from interstitial fibroblasts, epithelial to mesenchymal transition of type II alveolar epithelial cells, and the differentiation of recruited fibrocytes. There are many mechanisms that are utilized by these cells for survival, proliferation, and persistent activation including up-regulation of cytokines [i.e., Interleukin 6 (IL-6) and C-C motif chemokine ligand 21 (CCL21)], cytokine receptors [i.e., Interleukin 6 Receptor 1 (IL-6R1), Glycoprotein 130 (gp130) and C-C Chemokine Receptor type 7 (CCR7)], and innate pattern recognition receptors [(PRRs; i.e., Toll Like Receptor 9 (TLR9)]. In this review, we will discuss the role of the cytokines IL-6 and CCL21, their receptors and the PRR, TLR9, in fibroblast recruitment, activation, survival, and differentiation into myofibroblasts in IPF.

## INTRODUCTION

Interstitial lung diseases (ILD) are a heterogeneous group of non-neoplastic pulmonary disorders that affect 500,000 people in US and are thought to result from inflammatory and/or aberrant repair mechanisms in the lung (King, 2005). Idiopathic pulmonary fibrosis (IPF) is the most common clinical form of ILD, with poor prognosis, median survival at 3–5 years after diagnosis, and no effective pharmacological intervention (du Bois, 2010). IPF is characterized histologically by the presence of usual interstitial pneumonia (UIP), containing fibroblastic foci, which are believed to be the site of active tissue remodeling due to presence of activated fibroblasts. The fibrotic triggers in IPF are unknown but it is speculated that persistent lung injury leads to alveolar epithelial cell injury and death, and subsequent aberrant repair mechanism(s) ablates the alveolus (Hecker and Thannickal, 2011; Wynn, 2011; Wolters et al., 2013).

The circumstances leading to aberrant repair mechanisms in IPF are unknown. However, several reports document heterogeneous responses of IPF lung fibroblasts to several inflammatory and non-inflammatory stimuli. In this report, we will review work pertaining to the responses of IPF lung fibroblasts to the cytokines, CCL21 and IL6, and to the pathogen associated molecular pattern (PAMP), hypomethylated CpG DNA (hereon referred to as CpG-ODN), and its receptor TLR9.

## CCR7 AND CCL21

Cytokines have been shown to play a major role in the progression of pulmonary fibrosis in both mice and human. Chemokines are a class or chemotactic cytokines that bind to and activate their receptors to induce cellular activation and migration. Several chemokine and chemokine receptors are highly expressed in IPF lungs and are thought to play a role in fibrogenesis. To this end, several *in vitro, in vivo* and *ex vivo* studies have identified CCL21 and its receptor CCR7 as important players in the progression of lung fibrosis.

CCR7 is a member of the G protein coupled receptors that is activated by its ligands CCL19 [also called EBI1 ligand chemokine (ELC) and macrophage inflammatory protein 3 beta (MIP-3β)] and CCL21 [also called 6-Ckine, exodus-2, and secondary lymphoid-tissue chemokine (SLC)]. CCR7 is highly expressed on T lymphocytes and activated dendritic cells where it was shown to be important for the migration of these cells into lymphoid tissues, which express a high concentration of CCL19 and CCL21. Further, CCL21 has been implicated in the formation of secondary (i.e., lymph nodes) and tertiary (i.e., bronchoalveolar lymphoid tissues) lymphoid tissues, with the latter being observed in IPF lung biopsies (Campbell et al., 1985; Wallace et al., 1996; Marchal-Somme et al., 2006; Rangel-Moreno et al., 2006). CCR7 has been shown to be utilized by many tumor cells for survival and lymphatic metastasis [Reviewed in (Fang and Hwang, 2009)]. Recently the expression of CCR7 was observed to be significantly increased in the lungs of IPF patients (Choi et al., 2006) suggesting that this receptor might be involved in the genesis of IPF.

Gene expression studies performed by Choi et al. (2006) indicated that CCR7 mRNA levels were markedly increased in IPF as compared to normal lung biopsies in contrast to the CCR7 ligands, CCL19, and CCL21, which were similar in the two biopsies (Choi et al., 2006). Histological analysis confirmed the gene expression studies and indicated that CCR7 was focally expressed in IPF lungs but not in normal lung biopsies, where there were no detectable CCR7 staining. Further, CCR7 staining partially co-localized with CD45^+^ cells but not with alpha Smooth Muscle Actin (αSMA)^+^, Collagen I^+^ (COL1), or CD34^+^ cells suggesting that fibrocytes did not express this chemokine receptor in the analyzed tissues. Morphological analysis of the histologically stained sections suggested that CCR7 staining was present in fibroblasts, mononuclear, and epithelial cells (Choi et al., 2006). In a subsequent study, IPF fibroblasts expressed significantly greater levels of CCR7 as compared to normal lung fibroblasts, confirming the histological findings of Choi et al. (2006) and suggesting that these cells might contribute to the increased expression of CCR7 in IPF (Pierce et al., 2007a,b).

Under normal conditions, CCR7 is predominately expressed by T cells, B cells, and dendritic cells; however, its expression was also observed in several cancers and cancer cell lines where it is thought to promote cell survival and lymphatic metastasis. Similar to cancer cells, studies have suggested that IPF lung fibroblasts might utilize CCR7 for migration into the lungs, proliferation and survival. Pierce et al. (2007a) have shown that IPF fibroblasts can migrate to CCL21. Further, this migration was dependent on the both CCR7 and CCL21 as shown by the inhibition of migration after the immuno-neutralization of either of these proteins (Pierce et al., 2007a). This study showed that in addition to promoting cellular migration, CCL21 enhanced fibroblast proliferation, CCL5, and αSMA expression. This was hypothesized to be due to the enhanced activation of the Mitogen Activated Protein Kinase (MAPK) and Extracellular signal Related Kinase 1/2 (ERK1/2) pathways by CCL21 in IPF as compared to normal lung fibroblasts. In another study performed by the same group, lung fibroblasts were purified from normal and IPF lung biopsies, cultured and then intravenously injected into C.B-17SCID mice, where they were observed to localize to the lung of these mice and induce significant lung fibrosis 63 days after injection (Pierce et al., 2007b). Further, IPF fibroblasts induced a significant increase of CCL21, Cathepsin E, MMP-19 and TIMP-1 expression by other cells in the fibrotic lungs of these mice 63 days after injection. Immuno-neutralization of CCR7 or CCL21 significantly reduced fibrosis in the lungs of IPF fibroblasts injected C.B-17SCID mice. These studies suggest that IPF fibroblasts utilize CCR7 and CCL21 for migration, survival, proliferation and activation, in a similar manner as many cancer cells, and that targeting this chemokine and its receptor therapeutically might ameliorate IPF.

Several studies have shown that fibrocyte differentiation contributes to fibrosis in several fibrotic diseases by expression and secretion of Extracellular Matrix (ECM) proteins and differentiation into myofibroblasts, which further contributes to the accumulation ECM proteins. Fibrocytes are a population of cells with fibroblast like properties, characterized by their expression of CD45, CD34, and COL1 (Bucala et al., 1994). These cells are heterogeneous in nature; they were also reported to express COL3, vimentin, fibronectin, CD11b, CD80, CD86, and the chemokine receptors CCR2, CCR7, and CXCR4 (Bucala et al., 1994; Abe et al., 2001; Schmidt et al., 2003; Phillips et al., 2004). Fibrocytes were shown to migrate toward CCL21 in an *in vitro* transwell chemotaxis assay (Abe et al., 2001). Checkerboard analysis confirmed that this migratory response toward CCL21 was chemotactic but not chemokinetic. Further, following the intravenous injection of labeled fibrocytes in mice, intradermal injection of CCL21 induced the recruitment of fibrocytes to the site of injection, confirming the results observed in the *in vitro* transwell chemotaxis assay (Abe et al., 2001).

The role of fibrocytes in IPF is currently controversial; however, it has been observed that there is a significant increase in the number of circulating fibrocytes in IPF patients (Mehrad et al., 2007), suggesting that these cells might contribute to this disease. Using a murine model of lung fibrosis, Phillips et al. (2004) have identified several populations of fibrocytes based on their surface expression of the chemokine receptors CXCR4 and CCR7 in mice, CD45^+^ COLI^+^ CXCR4^+^ CCR7^-^, and CD45^+^ COLI^+^ CXCR4^-^ CCR7^+^. This study indicated that CXCR4^+^cells are the predominant population of fibrocytes recruited to fibrotic lungs of mice in a bleomycin model of lung fibrosis and that neutralizing CXCL12 significantly reduced lung fibrosis. This was further supported by other studies where it was shown that CXCR4^+^fibrocytes were the predominant population of fibrocytes in circulation in IPF patients and that these cells were present in histological sections from human IPF lung biopsies (Mehrad et al., 2007; Andersson-Sjöland et al., 2008). Further, a recent study have shown that there were no differences in the number of fibrocytes recruited into murine fibrotic lungs in response to bleomycin challenge between wild type and CCR7-/- mice further supporting that CCR7 might not be the predominant chemokine receptor required for the recruitment of fibrocytes into fibrotic lungs in mice (Trujillo et al., 2010a).

**FIGURE 1:**
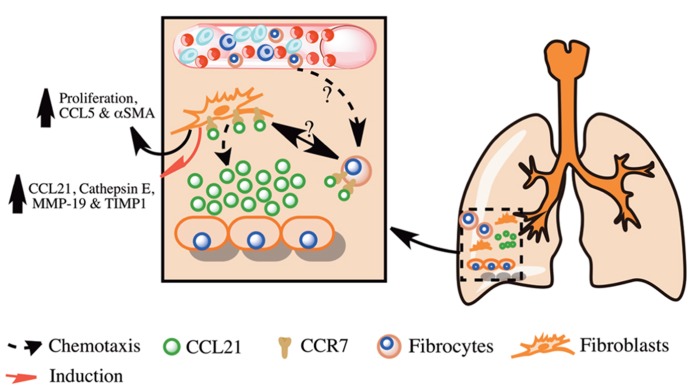
**Overview of the role of CCL21 and its receptor CCR7 in pulmonary fibrosis.** Shown is a simplified overview of the role of CCL21 produced in fibrotic lungs in the promotion of pulmonary fibrosis via the recruitment and activation of lung fibroblasts and fibrocytes.

Collectively, these studies suggest that CCR7 is expressed on IPF lung fibroblasts and is utilized for their activation, survival, and proliferation **Figure 1**. Further, CCR7 is expressed on fibrocytes but it is not the dominant chemokine receptor utilized by these cells for their recruitment in murine fibrotic lungs. These studies suggest that targeting CCR7 and its ligand CCL21 might lead to reduced fibroblast activation, proliferation, and survival in IPF and the amelioration of this disease.

## IL-6 AND IL-6Rα/GP130

IL6 is a multifunctional cytokine belonging to the IL-6 family of cytokines, which includes Interleukin 11 (IL-11), oncostatin M, ciliary neurotrophic factor, leukemia inhibitory factor, and cardiotrophin-1. The IL-6 family is characterized by signaling through a common broadly expressed receptor, gp130 (also known as IL6ST, IL6-beta, or CD130), which forms a heterodimer with a unique alpha chain, such as IL-6Rα for IL-6, to induce either pro-inflammatory or anti-inflammatory responses. Several members of the IL-6 family of cytokines, including IL-6, IL-11, and oncostatin M, have been implicated in IPF with the former being the best-studied member in this disease. *In vitro* studies indicated that IL-11 was mitogenic and inhibited Fas induced apoptosis of lung fibroblasts derived from normal and IPF lung biopsies (Moodley et al., 2003a,b). Other studies indicated that oncostatin M was significantly increased in bronchoalveolar lavage fluid (BALF) from IPF patients and in the lungs of bleomycin challenged mice; and its chronic intranasal administration induced a fibrotic response in murine lungs, which was independent from Interleukin 4 (IL-4)/Interleukin 13 (IL-13) and Transforming Growth Factor-beta (TGF-β) signaling (Richards et al., 2002; Mozaffarian et al., 2008). Further *in vitro* studies indicated that oncostatin M was mitogenic, inhibited spontaneous fibroblast apoptosis, induced COL expression by human lung fibroblasts and inhibited TGF-β induced αSMA expression by a subset of IPF lung fibroblasts characterized by the presence of constitutively active Signal Transducer and Activator of Transcription 3 (STAT3; Scaffidi et al., 2002; Pechkovsky et al., 2012). These studies suggest that this family of cytokines might contribute to the fibrotic response in IPF.

**FIGURE 2:**
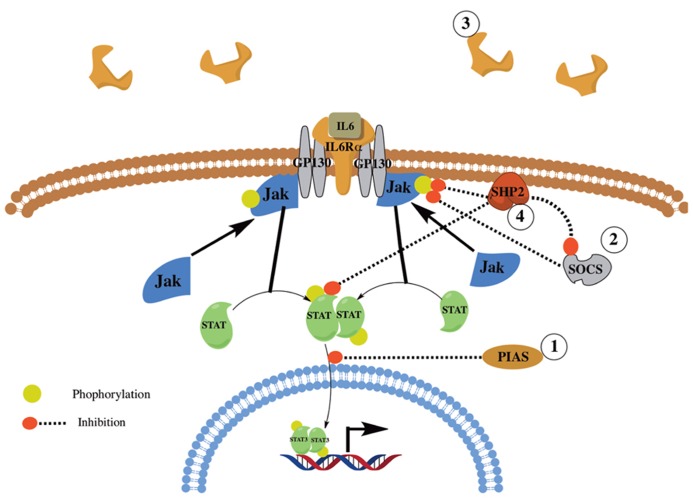
**Overview of IL6 signaling and regulation.** Shown is a simplified overview of IL6 signaling and regulation. IL6 signals through its receptor, IL6αR, and GP130. The signal is then transduced through JAK1/2 and STAT1/3 and the SHP2/ERK MAPK pathways (not shown). IL6 signaling is regulated via the action of several proteins and mechanisms including PIAS, SOCS, SHP2, and the shedding of IL6Rα.

Interleukin-6 is expressed and secreted by various cells including alveolar macrophages, lung fibroblasts (Shahar et al., 1996), and fibrocytes (Chesney et al., 1998). Upon binding to its receptor, IL-6 activates the Janus Kinase (JAK)/STAT; (i.e., JAK1/2 and STAT1/3) and the SH2 domain containing protein tyrosine phosphatase SHP-2 and ERK MAPK pathways [reviewed in (Kamimura et al., 2003)]. IL-6 signaling is tightly regulated by various proteins and mechanisms including: (1) Protein inhibitor of activated STAT (PIAS), which inhibits STAT activation; (2) Suppressor Of Cytokine Signaling (SOCS), which inhibits JAK signaling; and (3) Shedding of IL-6Rα to create a soluble decoy receptor. Further, SHP-2 has been shown to be a negative regulator, by dephosphorylating activated JAK and STAT proteins leading to reduced JAK/STAT signaling, and a positive regulator, by the inhibition of SOCS binding with JAK thus enhancing JAK/STAT signaling [**Figure 2**; reviewed in (Xu and Qu, 2008)].

Due to its multifunctional properties, there is great interest in dissecting the role of IL-6 in IPF. The concentration of IL-6 was observed to be significantly increased in BALF from IPF patients as compared to control BALF (Takizawa et al., 1997) suggesting that this cytokine might be involved in the progression of IPF. Further, IL-6 was expressed and secreted by alveolar macrophages and lung fibroblasts in response to TNF-α stimulation (Shahar et al., 1996). Interestingly, IL-6 inhibited proliferation in normal lung fibroblasts but was mitogenic to IPF lung fibroblasts (Moodley et al., 2003b). This was shown to be independent of gp130 expression and to be due to differential IL-6 signaling in these two fibroblast populations: in normal lung fibroblasts, IL-6 induced sustained STAT3 signaling leading to increased expression of the Cyclin Dependent Kinase Inhibitor (CDKI) p19^INK4D^; however, in IPF lung fibroblasts, IL-6 induced sustained SHP-2/ERK MAPK signaling leading to the decreased expression of the CDKI p27^Kip1^, increased Cyclin D_1_ activation, and enhanced proliferation (Knight et al., 2003).

In a subsequent study, IL-6 induced the resistance of IPF lung fibroblasts to Fas-induced apoptosis due to the increased expression of the anti-apoptotic protein BCL-2, in contrast to IL-6 stimulated normal lung fibroblasts, which were more sensitive to Fas-induced apoptosis due to the increased expression of the pro-apoptotic protein Bax (Moodley et al., 2003a). Interestingly, the increased BCL-2 and decreased Bax expression in IPF and normal lung fibroblasts, respectively, were shown to be dependent on STAT3 suggesting that STAT3 activation in these fibroblasts is differentially regulated (Moodley et al., 2003a). This was supported by another study, which indicated that there was a significant decrease in the expression of the JAK/STAT3 inhibitor, SOCS1, in IPF as compared to normal lung fibroblasts, which was correlated with increased COL1 expression by IPF lung fibroblasts (Shoda et al., 2007). A role of SOCS1 in lung fibrosis was supported by another study using SOCS1-haplodeficient (+/-) mice in a bleomycin induced model of lung fibrosis (Nakashima et al., 2008). In this study, bleomycin challenged SOCS1-haplodeficient mice developed significantly increased pulmonary fibrosis as compared to wild type mice further supporting that altered gp130 signaling can exacerbate a fibrotic response.

Recently, Pechkovsky et al. (2012) identified a population of fibroblasts from IPF lung biopsies which were characterized by the constitutive activation of STAT3. These fibroblasts had lower proliferation rates, decreased αSMA, Thy-1, and β_3_ integrin expression as compared to normal lung fibroblasts and were not localized in αSMA rich fibroblastic foci, but were localized near areas of dense fibrosis. This study suggests that there are different populations of fibroblasts present in the fibrotic lungs in IPF, and that these populations respond differently to IL-6/gp130 signaling. To determine the effect of hyperactive STAT3 signaling and IL-6 on lung fibrosis, an elegant study was performed using transgenic mice containing a mutation in the common IL-6 family receptor subunit, gp130, leading to polarized and dysregulated STAT1/3 (gp130^757F^), or Erk1/2 (gp130^ΔStat^) signaling upon receptor activation (O’Donoghue et al., 2012). When challenged with bleomycin, the gp130^757F^ transgenic mice exhibited an increase in lung fibrosis, COL1, and IL-6 expression when compared to wild type, bleomycin-challenged mice. Also, the knockdown of IL-6 (i.e., gp130^757F^; IL-6-/-) ameliorated the observed fibrotic response in these mice. Further, the pro-fibrotic role of IL-6 and its signaling pathway was examined *in vivo* using the bleomycin model of lung fibrosis in an IL-6-/- mouse (Saito et al., 2007). In this study, there was a significant reduction in bleomycin induced lung fibrosis in the IL-6-/- mouse as compared to wild type mice.

Collectively, these studies indicate that IL-6 and IL-6 receptor signaling play an important role in the fibrotic response in IPF and that there is a heterogeneous population of fibroblasts with skewed IL-6 signaling in IPF lungs leading to the pro-fibrotic and anti-apoptotic responses by these cells.

## TLR9

The innate immune system plays a major role as a first line of defense against many pathogens by their sequestration and/or destruction and clearance followed by the initiation of a regenerative healing response. To function rapidly and efficiently, the innate immune system utilizes a class of receptors, called Pattern Recognition Receptors (PRRs) to sense and react to proteins, lipids, and nucleic acids released from the damaged host or activated inflammatory cells (Damage Associated Molecular Pattern or DAMPs) or by the infecting pathogen (Pathogen Associated Molecular Pattern or PAMPs). Currently, 10 Toll Like Receptors (TLRs) have been identified in human (TLR 1-10), and 12 in mice (TLR 1–9 and 11–13) with TLR 1–9 being the best characterized. TLR1, 2, 4, 5, and 6 are expressed on the plasma membrane and TLR 3, 7, 8, and 9 are predominantly expressed on the membrane of the endoplasmic reticulum (ER) and are translocated to endosomal membranes upon cellular activation where they bind to microbial RNA and DNA. Most TLR ligands induce the homodimerization [TLR 3, 4, 5, 6, 7 (in mouse), and 9] or heterodimerization (TLR1/ 2, TLR6/2, and TLR7/8 in human) of TLRs to induce a cellular response.

Toll Like Receptor 9 is a member of the TLR family of PRR, which recognizes hypo-methylated CpG containing DNA from bacteria (Hemmi et al., 2000), viruses (Lund et al., 2003), and mitochondria from damaged host cells (Zhang et al., 2010) and is predominantly expressed by immune cells. It is primarily localized in the membrane of the ER and is translocated into endosomes upon cellular activation, where its cytoplasmic domain is proteolytically modified by acid-dependent proteases to generate a signaling receptor (Ewald et al., 2011).

Idiopathic pulmonary fibrosis is characterized by the sustained activation of myofibroblasts and excessive deposition of ECM proteins leading to the loss of the normal lung architecture. It is thought that a low-grade chronic inflammatory response might contribute to the activation and proliferation of myofibroblasts and the enhanced fibrotic response in IPF and thus there is great interest in the role of PRR receptors such as TLR3 and TLR9 in this disease. IPF has been previously observed to progress at different rates, with some patients showing slow progression characterized by the gradual decline of lung functions (slow progressors) and other patients progressing at a faster rate with a rapid decline of lung functions (rapid progressors). Recently, TLR9 expression was shown to be higher in untreated IPF as compared to normal lung fibroblasts and treatment of IPF but not normal lung fibroblasts with the Th2 cytokines IL-4 and IL-13 further increased its expression (Meneghin et al., 2008). Further, fibroblasts derived from rapid progressor IPF lung biopsies were observed to have higher response to the TLR9 agonist, CpG-ODN, as compared to fibroblasts derived from slow progressor IPF lung biopsies (Trujillo et al., 2010b), suggesting that TLR9 signaling is enhanced in rapid progressor fibroblast and that this PRR might contribute to the enhanced progression of IPF in rapid progressor IPF patients. TLR9 activation by CpG-ODN treatment induced the differentiation of IPF but not normal lung fibroblasts into myofibroblasts as evident by increased αSMA expression (Meneghin et al., 2008), induced the differentiation of TGF-β treated blood monocytes into CD45^+^ αSMA^+^ COL1^+^ fibrocytes, and induced the Epithelial to Mesenchymal Transition (EMT) of the type II alveolar epithelial cell line, A549 cells (Trujillo et al., 2010b).

Recently, it was shown that there was a defect in miRNA processing in rapid progressor lung fibroblasts which the authors hypothesized to be due to decreased expression of two proteins, Argonaute RISC catalytic component 1 and 2 (AGO1 and AGO2, respectively), which are essential components of the RISC complex (Oak et al., 2011). In a subsequent study, CpG-ODN treatment was observed to significantly reduce AGO1 expression in rapid progressor lung fibroblasts, further supporting a role of TLR9 activation in the aberrant profibrotic phenotype of IPF fibroblasts from rapid progressor IPF patients (Hogaboam et al., 2012).

The role of TLR9 in fibrosis was further studied *in vivo* using mouse models of lung fibrosis. By intravenous injection of human lung fibroblasts into C.B-17SCID mice, (Trujillo et al., 2010b) have shown that fibroblasts isolated from rapid progressor IPF lung biopsies increased the fibrotic response in the lung 63 days after injection as compared to fibroblasts isolated from slow progressor IPF lung biopsies. Further, intranasal treatment with CpG-ODN induced a significant increase in lung fibrosis in the rapid progressor but not the slow progressor fibroblasts injected mice further supporting the *in vitro* studies showing increased pro-fibrotic responses by these fibroblasts in response to CpG-ODN treatment. In another model of lung fibrosis, it was observed that the bleomycin induced lung fibrosis was similar between TLR9-/- and wild type mice; however, when bleomycin challenged wild type mice were intranasally administered with CpG-ODN, there was a reduction in lung fibrosis as compared to untreated bleomycin challenged wild type mice (Luckhardt et al., 2011). In this study, the authors hypothesized that the observed reduction in lung fibrosis in response to CpG-ODN treatment is due to the increased TLR9 induced Interferon-beta (IFN-β) expression, which was shown to reduce lung fibroblast proliferation. These studies suggest that TLR9 responses in bleomycin-induced fibrosis, a model which is characterized by a strong inflammatory driven fibrotic response, are different from those observed in humanized SCID model of pulmonary fibrosis, a model which is primarily driven by the injected human fibroblasts and is largely independent from any inflammatory responses, and that the role of TLR9 in fibrosis might be dependent on the surrounding cytokine environment as evident by the different TLR9 induced response observed in the inflammatory dependent and the inflammatory independent murine pulmonary fibrotic models.

Collectively, these studies suggest that TLR9 expression is enhanced in lung fibroblasts derived from rapid progressor IPF lung biopsies and the activation of this receptor leads to increased fibrotic response of human lung fibroblasts, the differentiation of monocytes into fibrocytes, and the EMT of alveolar type II cells leading to dysregulated ECM production and the deterioration of the normal lung architecture. However, future studies are warranted to dissect the signaling pathways activated in IPF versus normal lung fibroblasts to CpG-ODN stimulation and the role of other nucleic acid sensors in the response of these fibroblasts to microbial nucleic acids.

## Conflict of Interest Statement

The authors declare that the research was conducted in the absence of any commercial or financial relationships that could be construed as a potential conflict of interest.
